# Spiritual Intelligence: A Scoping Review with Concept Analysis on the Key to Spiritual Care

**DOI:** 10.3390/jintelligence14020024

**Published:** 2026-02-03

**Authors:** Cristina Teixeira Pinto, Ângela Coelho, Lúcia Guedes, Rui Nunes, Sara Pinto

**Affiliations:** 1 Faculty of Medicine, University of Porto, 4200-319 Porto, Portugal; 2 Intrahospital Palliative Care Team, Unidade Local de Saúde de Entre Douro e Vouga, 4520-211 Santa Maria da Feira, Portugal; 3 RISE-Health, MEDCIDS—Department of Community Medicine, Information and Health Decision Sciences, Faculty of Medicine, University of Porto, 4200-319 Porto, Portugal

**Keywords:** concept analysis, intelligence, spiritual intelligence, spirituality, holistic health, spiritual care

## Abstract

This study explores the concept of spiritual intelligence from an evolutionary perspective, providing a comprehensive and updated definition. A concept analysis was conducted following Rodgers’ Evolutionary Method, supported by a scoping review in accordance with the Joanna Briggs Institute methodology. Studies explicitly addressing spiritual intelligence, regardless of population, setting, or discipline, were included across quantitative, qualitative, mixed-methods, and review designs. Four databases—PsycINFO, PubMed Central, Scopus, and Web of Science—were searched for publications up to 15 December 2025, using the term “spiritual intelligence.” One-hundred-twelve articles met inclusion criteria and were analyzed through narrative synthesis and inductive analytical processing. Spiritual intelligence emerged as a construct encompassing adaptive cognition, higher consciousness, problem management, and personal growth, often referred to as Spiritual Quotient or Existential Intelligence. Antecedents included self and transcendental awareness, existential questioning, and search for meaning and purpose, while consequents comprised enhanced health, performance, self-awareness, and humanitarian orientation. Defining attributes were equanimity, life-wisdom, transcendental awareness, spiritual consciousness, meaning and purpose creation, and existential questioning. This evolutionary analysis traced the concept from theory to application, revealing its positive influence in daily life. Equanimity and life-wisdom were identified as core attributes, highlighting implications for training and integration of spiritual care in professional practice.

## 1. Introduction

The foundational concepts of cognitive intelligence (Howard, 1993) and emotional intelligence (Salovey & Mayer, 1990) have profoundly shaped our understanding of human intellect. However, while these traditional frameworks effectively illuminate our analytical ability and interpersonal skills, they may not fully encompass the depth of human experience or the innate search for meaning and purpose (Checa & Fernández-Berrocal, 2015). Thus, a broader perspective on intelligence is needed: one that encompasses not only ‘how’ we think and relate, but also ‘why’ we seek meaning and purpose. This has led to the exploration of spiritual intelligence as a distinct dimension of human intelligence.

Although definitions vary, studies applying the concept commonly describe spiritual intelligence as the ability to integrate spiritual or existential beliefs into daily functioning. For example, Babanazari et al. (2012) help understand spiritual intelligence as a meaning-making capacity that supports coping and psychological adjustment; Ajele et al. (2021) explore it as a set of values-based competencies that enhance comfort and well-being; and Amirian and Fazilat-Pour (2016) describe spiritual intelligence as the ability to apply spiritual principles to personal and interpersonal daily life challenges. These applications illustrate how spiritual intelligence is understood and operationalized across contexts, highlighting its relevance for expanding traditional models of intelligence and enriching approaches to human well-being and care.

Since the late nineties there has been a growing recognition of spirituality as an important source of adaptability and improved mental performance through many quantitative studies acknowledging the impact of spiritual intelligence in enhancing various daily life interactions as well as being a trainable asset for better mental and general health (Pinto et al., 2024). This evolution in our understanding has prompted the scientific community to conceptualize spiritual intelligence as a distinct dimension of human intelligence relevant not only to our psychological well-being, but also to practical aspects of daily life, including the provision and experience of care (Aliabadi et al., 2021; Ghalaychi et al., 2019; Kaur et al., 2013; Riahi et al., 2018). Indeed, as contemporary healthcare increasingly emphasizes person-centered approaches (Aliabadi et al., 2021; Pinto & Pinto, 2020), integrating spiritual intelligence into care practices offers meaningful opportunities to enhance comfort, coping, and overall well-being.

By recognizing and actively fostering spiritual intelligence, healthcare professionals, patients, and informal caregivers can build more empathetic, resilient, and purpose-driven therapeutic relationships (Aliabadi et al., 2021; Kaur et al., 2013). Practical efforts to advance the conceptualization and measurement of spiritual intelligence therefore hold significant promise for improving both the quality and the humanity of healthcare delivery.

## 2. Background

The discussion of spiritual intelligence emerges from the early premise that one’s spiritual resources contribute to better daily functioning (Emmons, 2000). With this in mind, different authors began to conceptualize this capability within the multiple intelligence model perspectives (Gardner, 1983). This approach positions spiritual intelligence as an intelligence focused on the ability to engage with matters of meaning, values and purpose (Zohar & Marshall, 2000), and to explore existential questions about life and interconnectedness (Noble, 2001), while also integrating spiritual aspects into cognitive and emotional daily life matters (Vaughan, 2002; Wolman, 2001), and promoting personal growth, self-awareness, compassion and integrity (Wigglesworth, 2012).

Further insight on the spiritual intelligence concept brought along more structured frameworks with different dimensions or themes attributed to this kind of intelligence, such as critical existential thinking, personal meaning production, transcendental awareness (as in meta-awareness and a sense of interconnectedness) and conscious state expansion (as in heightened states of awareness), proposed by (King & DeCicco, 2009), or consciousness, grace, meaning, transcendence, truth, serenity and interconnectedness, suggested by (Amram & Dryer, 2008), amongst other perspectives.

Although spiritual intelligence is frequently discussed within spiritual or religious contexts, it is important to clearly differentiate it from spirituality, religion, and emotional intelligence. Pinto et al. highlight that spirituality refers broadly to personal beliefs, values, and existential meaning-making, while religion involves structured traditions, doctrines, and communal practices (Pinto et al., 2024). Emotional intelligence, in contrast, concerns the recognition and regulation of emotions in oneself and others, facilitating interpersonal relationships (Salovey & Mayer, 1990). Spiritual intelligence is distinguished from all three by its focus on the capacity to derive meaning, purpose, and value-based insight from all lived experiences and existential questions, enabling individuals to integrate these insights into decision-making and personal growth, thus conciliating spirituality with daily life experiences for better adaptability (Pinto et al., 2024).

Despite many theorists clarifying spiritual intelligence as a distinct, although related, concept from spirituality and its independence from religious affiliation or practices, it is frequent the misuse of terminology and the connection of spiritual intelligence to religiosity, conceptualizing it as the capacity to comprehend religious issues (Saeedi et al., 2014) to relate to one’s deity (Anwar et al., 2020) or to utilize religious and spiritual patterns in everyday life (Imani et al., 2021).

These inconsistencies reinforce the need to further clarify the spiritual intelligence concept to guarantee its intercultural universality.

With spiritual intelligence being a developing, multidimensional concept, this evolutionary perspective is pivotal to analyzing its history as well as its current state and ongoing challenges across different cultural and disciplinary contexts, while also pointing toward future research directions that deepen our understanding of this complex human capacity.

Over the past two decades, interest in spiritual intelligence has grown steadily in education, health and psychology, but existing models still diverge in how they specify its core attributes, distinguish it from related constructs (e.g., spirituality, religiousness, personality) and justify its status as an ‘intelligence’. At the same time, empirical studies increasingly associate spiritual intelligence with resilience, coping and mental health (Ajele et al., 2021; Hatami et al., 2019; Kaur et al., 2013; Khosravi & Nikmanesh, 2014; Narayanan & Jose, 2011; Parattukudi et al., 2021; Pinto et al., 2024; Polemikou & Vantarakis, 2019), which has intensified theoretical debate while leaving key conceptual issues unresolved, with new attributes, applications, and theoretical frameworks emerging since the initial proposed definitions. This has led to conceptual fragmentation and terminological drift, underscoring the need for an updated, evidence-based synthesis.

Building on this understanding, this study aims to provide a comprehensive and updated definition of spiritual intelligence based on its historical trajectory and contemporary patterns of use as well as on its cultural and contextual variability, thereby enhancing future research and measurement initiatives. More specifically, it aims to explore the concept’s evolution in recent years to propose a refined conceptualization, one well-suited for assessing spiritual intelligence as an outcome.

## 3. Data Sources

We conducted a concept analysis study using Rodgers’ Evolutionary Method (Rodgers & Knafl, 2000), an approach that views concepts as dynamic entities shaped by context and time. This inductive method systematically explores how a concept is used and understood in the literature, emphasizing its development rather than seeking a fixed definition. The method involves identifying key attributes, antecedents, consequences, related terms, and contextual variations, thereby allowing for a comprehensive understanding of the concept’s evolution and current applications (Rodgers & Knafl, 2000).

To operationalize Rodgers’ method, we specifically conducted a scoping review following the Joanna Briggs Institute methodology for scoping reviews (Peters et al., 2020). A scoping review is methodologically aligned with Rodgers’ evolutionary concept analysis because it enables comprehensive mapping of a concept’s attributes, antecedents, and consequences across diverse contexts. Its flexible yet rigorous structure allows the inclusion of empirical, theoretical, and gray literature, supporting the exploration of conceptual boundaries and historical changes over time.

A scoping review methodology was preferred over a systematic review since the strength of the results were not the main aim of our study and it would likely exclude relevant theoretical papers that are important in clarifying the concepts’ evolution. A narrative review, on the other hand, would not grant the methodological transparency and reproducibility that Rodger’s method requires. Thus, by capturing the breadth, variability, and evolution of the spiritual intelligence concept, while maintaining transparent methods, this scoping review enhances both the rigor and the exploratory nature required by Rodgers’ framework. Data were reported according to the PRISMA extension for scoping reviews (PRISMA-ScR) (Page et al., 2021).

We included studies involving participants of any age, background, or setting. To ensure conceptual clarity, only studies that explicitly addressed spiritual intelligence were selected. Studies that focused solely on general spirituality, without direct reference to spiritual intelligence, were excluded. We considered literature from a broad range of contexts and disciplines, including but not limited to psychology, healthcare, and education, to capture the diversity of settings in which the concept is applied.

Regarding study types, we included quantitative studies that employed validated instruments for assessing spiritual intelligence and presented reproducible methodologies, reviews, qualitative, and mixed-methods studies. Editorials, conference abstracts, theoretical papers, and studies lacking sufficient methodological detail were excluded.

### 3.1. Search Strategy and Study Selection

We conducted a comprehensive search across four databases: PsycINFO, PubMed Central, Scopus, and Web of Science, using the search term ‘spiritual intelligence.’ The search strategy was tailored to each database and limited to studies published in English, French, Portuguese or Spanish, up to 15 December 2025.

After the initial search, all references were imported into Mendeley^®^ for deduplication. Two independent reviewers screened titles and abstracts against the established inclusion criteria. Following this, three reviewers conducted a thorough review of the full-text. Disagreements were resolved through discussion or with a third reviewer.

### 3.2. Data Extraction and Analysis

Data were extracted from the included studies using a predesigned extraction form developed by the review team. We independently extracted key study characteristics, such as authorship, year of publication, country, participant demographics, study settings, objectives, methodologies, and the instruments used to assess spiritual intelligence, including their reported reliability and validity. We also collected findings relevant to the concept of spiritual intelligence, focusing on how it was defined, measured, and contextualized.

Following data extraction, we performed a narrative synthesis of the collected information, guided by Rodgers’ Evolutionary Method of concept analysis (Rodgers & Knafl, 2000). This inductive analytical process involved systematically identifying and clustering the concept’s defining attributes, examining its antecedents (events or factors preceding the concept’s occurrence), and consequences (outcomes or effects of the concept) as described in the literature. We also analyzed surrogate terms and contextual variations that indicated how the concept’s understanding or application shifted across different disciplines, populations, or over time. The final step involved formulating a refined conceptualization of spiritual intelligence, incorporating these essential elements and accounting for relevant contextual factors, thereby providing a definition well-suited for its assessment as an outcome.

To further consolidate the data initially extracted and inductively coded by three independent human reviewers, we incorporated an artificial intelligence (AI)-assisted screening of the selected studies (Bennis & Mouwafaq, 2025). Specifically, a Large Language Model was used to perform a confirmatory analysis following the initial manual coding. This triangulated approach prioritized the nuanced insights of human interpretation, while harnessing the breadth and efficiency of AI. Any novel or divergent codes generated by the AI were systematically reviewed and discussed by two human reviewers. In cases of persistent disagreement or uncertainty regarding these AI-generated codes, a third reviewer was consulted to reach consensus. This process aimed to enhance the robustness, transparency, and validity of the final dataset.

All conceptual terms reported in this analysis emerged inductively from the included literature. While some interpretive synthesis was required to compile how these attributes were organized and articulated, no attribute was introduced by the authors alone; each reflects patterns of meaning, language, or conceptual emphasis present across the analyzed sources.

## 4. Overview of the Concept

**Figure 1:**
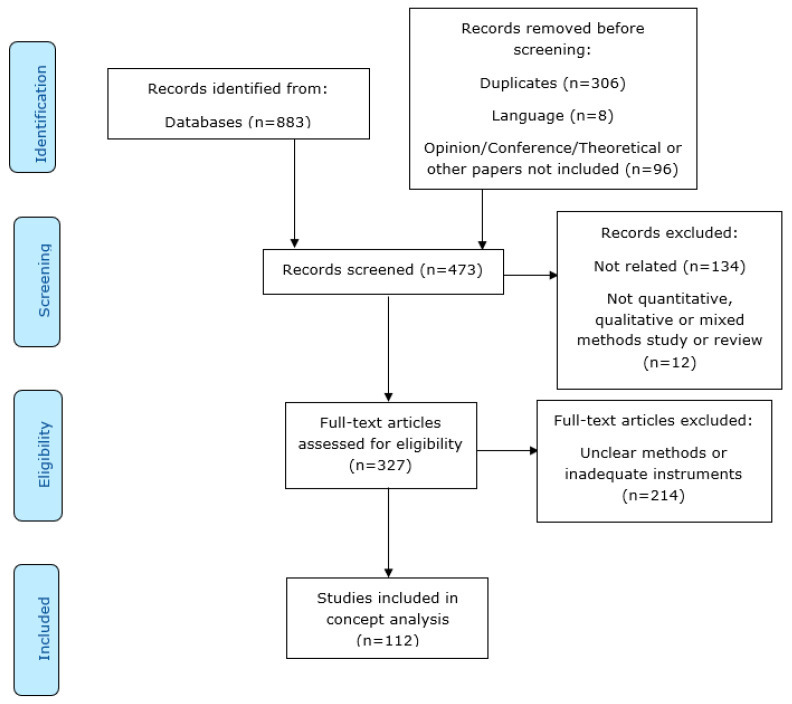
PRISMA-ScR flow diagram (Page et al., 2021).

Our searches of electronic databases identified 883 hits excluding duplicates. After screening, 473 hits were excluded, which identified 327 eligible studies. This resulted in 112 included studies (Supplementary Table S1). The results of this search and study inclusion process were reported according to the PRISMA-ScR flow diagram (Figure 1) (Page et al., 2021).

The included studies (Table 1) were published between 2005 and 2025 and originated predominantly from geographical areas where spirituality is deeply rooted into the social system and daily life, such as the Middle-East (*n* = 53), Asia (*n* = 19) and Europe (*n* = 19), despite the original theoretical works on the spiritual intelligence concept originating mainly from North America (Emmons, 2000; King & DeCicco, 2009; Noble, 2001; Vaughan, 2002; Wigglesworth, 2012; Wolman, 2001).

Most studies were quantitative (*n* = 94) and investigated spiritual intelligence in different settings, such as healthcare settings (*n* = 46, predominantly addressing spiritual intelligence in nurses and patients), universities (*n* = 24, mainly focusing on students and some also including employees), schools/education (*n* = 12, evaluating both students and teachers), or studies open to the general population (*n* = 19) aiming to broadly characterize or validate spiritual intelligence measuring instruments.

In the selected quantitative studies, David King’s (King & DeCicco, 2009) Spiritual Intelligence Self-Report Inventory—24 items were the most widely used instrument for measuring spiritual intelligence (*n* = 61).

### 4.1. References of the Concept and Areas of Application

The references of a concept concern the contexts in which it has been applied (Rodgers & Knafl, 2000). From the large variety of study settings described in Table 1, going from education to healthcare, organizations to sports or even military, one can recognize the wide usage of spiritual intelligence in daily life.

After close to a decade of theoretical development of the spiritual intelligence concept, the first quantitative studies started to take place (King et al., 2012; King & DeCicco, 2009; Martin & Hafer, 2009), initially still in the academic setting, targeting students and general population for better characterization of baseline spiritual intelligence and measuring instruments’ validation but as the potential benefit of spiritual intelligence in mental health started to raise interest, studies in healthcare settings and aiming to study high-burdened professions as nursing or military workers soon followed, concentrating on the resilience, stress and spiritual coping benefits of spiritual intelligence.

The healthcare setting kept a strong interest in spiritual intelligence throughout time, expanding the research of its implications not only for the healthcare workers but also for patients with different health requirements (diabetics (Ajele et al., 2021; Rahmanian et al., 2018, 2019; Rafiei et al., 2023), coronary heart disease patients (Nekouei et al., 2014), pregnant women (Abdollahpour & Khosravi, 2018; Hatami et al., 2019), recovering addicts (Shahbakhsh & Moallemi, 2013; Rajabi et al., 2023), amongst others) centering on spiritual intelligence’s potential towards better health outcomes, self-care and quality of care. With the COVID-19 pandemic’s deep impact on the healthcare system raising important ethical dilemmas and mental health issues amongst healthcare professionals as well as patients, this interest grew significantly in the aftermath (Zamani et al., 2023; Yadollahpour et al., 2023; Maghool et al., 2023).

With growing evidence on spiritual intelligence’s impact on communication and relational skills (Geram, 2016; Mosavinezhad et al., 2019; Mehralian et al., 2023), the study settings progressed to explore organizations (especially focusing on the benefits spiritual intelligence might bring to leadership and employee commitment (Dargahi & Veysi, 2021; Khastavaneh et al., 2018)) and the educational domain (studying spiritual intelligence impact on teachers’ and students’ teaching/learning strategies and dealing with the work/school stressors (Abdolrezapour & Alipour, 2021; Pishghadam et al., 2022; Singla et al., 2021)).

Thus, different areas of exercise of spiritual intelligence were studied across various populations all around the world and diverse cultural settings and religious beliefs, as reported on Table 1, contributing to a deeper and better understanding of spiritual intelligence through time and a growing impact of this concept concerning health care, educational and organizational development and practices as well as policy making.

Our analysis identified as major areas in which spiritual intelligence can be exploited: adaptative cognition processes, achieving elevated states of consciousness, problem management, personal growth, nurturing a humanitarian character, existentialism thinking, interpersonal relationships, mental health and work performance. Further details on the analysis of spiritual intelligence applications are described in Supplementary Table S2.

### 4.2. Concept Evolution

From its early conceptualizations, the understanding of spiritual intelligence concept has evolved significantly. Since the majority of the initial theoretical work on spiritual intelligence did not comply with our study inclusion criteria, we provide an overview of the concept evolution throughout this period.

Considering a multiple intelligences model approach to cognition (Gardner, 1983), spiritual intelligence advocates attempted to extend models of human cognition beyond traditional cognitive and emotional domains. At the turn of the century, several authors independently proposed that spirituality-related capacities might constitute a distinct domain of intelligence. Zohar and Marshall framed spiritual intelligence as the ability to integrate rational and emotional processes to address existential questions and derive meaning from experience; despite needing voluntary intellectual processing, spiritual intelligence highly relies on personal beliefs and experiences, more than linguistic or logical data (Zohar & Marshall, 2000). In the same period, Emmons argued that spiritual intelligence should be understood as an adaptive problem-solving capacity that uses spiritual information to enhance functioning (Emmons, 2000). Shortly thereafter, Vaughan emphasized transcendental awareness and higher levels of consciousness as core components of the construct, further expanding its scope (Vaughan, 2002).

Later, King & DeCicco proposed a 4-dimensional model of spiritual intelligence comprising critical existential thinking, transcendental awareness, personal meaning production and conscious state expansion, contemplating the major dimensions previously addressed (King & DeCicco, 2009).

As the field progressed, researchers sought to clarify how spiritual intelligence differed from and interacted with other forms of intelligence. King and DeCicco further conceptualized spiritual intelligence as enabling a compassionate and values-driven response to others’ distress, thus transcending the emotional intelligence’s action-limited ability to empathetically mirror emotions (King & DeCicco, 2009).

Expanding on this, Wigglesworth proposed an equanimous and humanitarian demeanor as key expressions of spiritual intelligence, emphasizing the ability to keep inner and outer serenity and remaining calm, wise and compassionate in any given circumstance (Wigglesworth, 2012).

Taken together, these contributions illustrate an evolving concept that has moved from initial meaning-making and existential framing toward a broader, more operationalized understanding encompassing awareness, compassion, adaptive functioning, and integrative consciousness.

### 4.3. Surrogate Terms

Surrogate terms present as alternative terminology for a given concept (Rodgers & Knafl, 2000). In our analysis, spiritual intelligence was frequently referred to as spiritual quotient or existential intelligence, linking to the inherent cognitive process involved and the underlying multiple intelligence framework (Gardner, 1983).

Also, expressions as spiritual well-being were used, conveying the connection between spiritual intelligence and better life adjustment. A state of heightened consciousness was another of the identified surrogate terms, relating to the aspects of consciousness and awareness strongly associated with spiritual intelligence. Further details on surrogate terms analysis are described in Supplementary Material Table S3.

### 4.4. Related Concepts

Related concepts are frequently associated with the studied concept, usually sharing some common attributes (Rodgers & Knafl, 2000).

Spirituality, or in a broader sense, the spiritual experience or expertise is the most related concept to spiritual intelligence, its intimate connection coming from the premise that spiritual intelligence draws from spirituality and self-consciousness and by combining it with deep cognitive processes that connect multiple intelligences achieves better personal outcomes in different life domains, such as health, performance, interpersonal relationships and organizational development.

For full details on related terms analysis refer to Supplementary Material Table S4.

### 4.5. Antecedents of the Concept

The antecedents of a concept are the premises in which it lays, the conditions required for it to occur (Rodgers & Knafl, 2000).

Deriving from the spiritual intelligence conceptualizations proposed in the various articles on our review, it is widely consensual that for spiritual intelligence to develop, it is required that the individual is connected to his spiritual-self and develops self and transcendental awareness, this is usually associated to a deep concern for existential questions and a search for meaning and purpose that derive from higher cognition. Spiritual intelligence also reflects self-investment and emotional awareness, usually associated with a humanitarian alignment.

Antecedents’ full analysis is described in Supplementary Material Table S5.

### 4.6. Consequents of the Concept

Consequents are the byproducts of a concept, the outcomes of its application and development (Rodgers & Knafl, 2000).

Since most of the selected studies in this concept analysis were quantitative correlational studies (Table 1) with proper quality assessment of methodology, they provide invaluable evidence on the consequents of spiritual intelligence known to date and open way for future research that may add to the concept’s evolutionary process further ahead.

Spiritual intelligence development and application was associated with better general and especially mental health (Ajele et al., 2021; Amirian & Fazilat-Pour, 2016; Bhandari et al., 2023; Dami et al., 2019; Nekouei et al., 2014; Parattukudi et al., 2021, better work performance (Ahmadi et al., 2021; Imani et al., 2021; Rahmawaty et al., 2021), humanitarian orientation (Kaur et al., 2013; Riahi et al., 2018), deep self-consciousness (Hojat & Badiyepeymaiejahromi, 2021; Rahmanian et al., 2018), a meaning and purposeful life (Babanazari et al., 2012; King & DeCicco, 2009), better organizational development (Alamanda et al., 2021; Anwar & Gani, 2015; Dargahi & Veysi, 2021; Singla et al., 2021) and interpersonal relationships (Aliabadi et al., 2021; Geram, 2016; Rahmawaty et al., 2021), and better intellectual performance (Gera et al., 2021) and problem management (Imani et al., 2021).

Detailed consequents’ analysis available in Supplementary Material Table S6.

### 4.7. Defining Attributes

In order to achieve a revised definition of spiritual intelligence, it is essential to first assess its key attributes, the main characteristics that define and distinguishes the concept (Rodgers & Knafl, 2000).

From the inductive analysis of the selected studies, we were able to produce, by a final interpretative synthesis process of the gathered data, six main domains for spiritual intelligence: equanimity, life wisdom, transcendental awareness, spiritual consciousness, meaning and purpose creation, and existential questioning.

Spiritual intelligence is characterized by several key aspects. Firstly, it encompasses equanimity, reflecting the strong inner-coherence, integrity, and inner-balance that allows individuals to remain calm, wise, and compassionate in any given circumstance. This attribute was consistently reported in studies examining resilience and emotional regulation in spiritual contexts (Ajele et al., 2021; Hatami et al., 2019; Khosravi & Nikmanesh, 2014; King & DeCicco, 2009; Martin & Hafer, 2009; Polemikou et al., 2019).

Another crucial facet is life wisdom, or the ability to navigate struggles with perseverance and a humanitarian approach to problems. Evidence for this attribute emerged mainly from studies describing reflective problem-solving and ethical decision-making (Amirian & Fazilat-Pour, 2016; Babanazari et al., 2012).

Furthermore, spiritual intelligence involves transcendental awareness, concerning the capacity to perceive what lies beyond oneself, including the ability to ascend to different levels of consciousness and attain a holistic or meta-view. Several studies highlighted participants’ experiences of connectedness and elevated consciousness (Ajele et al., 2021; King & DeCicco, 2009).

It also incorporates spiritual consciousness, which enables higher cognition and the intellectual integration of spiritual experiences, as supported by research emphasizing reflective and meaning-contemplation processes (Ajele et al., 2021; Geram, 2016).

The creation of meaning and purpose is another vital element, both from daily life experiences and in a long-term perspective of life goals, being widely reported in multiple studies (Babanazari et al., 2012; Badrudin et al., 2021; King & DeCicco, 2009; Parattukudi et al., 2021).

Finally, spiritual intelligence pertains to the ability for deep existential questioning and a profound concern for knowledge, also a recurrent theme across literature (Badrudin et al., 2021; King & DeCicco, 2009; Polemikou et al., 2019).

Further details on the attributes analysis are described in Supplementary Material Table S7.

### 4.8. Model Case

According to Rodgers’ evolutionary approach to concept analysis (Rodgers & Knafl, 2000), a model case serves to concretely illustrate the defining attributes of the concept in real life and that have been identified through the analysis process.

Mrs. Diana is a family caregiver supporting her husband through advanced illness within an interdisciplinary palliative care setting. Her journey provides a clear, concrete illustration of spiritual intelligence woven into daily care and collaboration with nursing and medical professionals.

Mrs. Diana demonstrates equanimity and calm, particularly in moments when her husband becomes more anxious or when unexpected symptoms arise. For example, when he feels more anxious, Mrs. Diana sits by his side, maintains a gentle tone, and takes a few deep breaths together with him before seeking help from the palliative care team. Nurses and physicians notice that she listens first, expresses understanding, and avoids reacting with panic, thus fostering a reassuring atmosphere even during periods of crisis.

She acts with integrity and purposeful communication, sharing her husband’s values and preferences with the team and ensuring these are reflected in the ongoing integrated individual care planning.

Mrs. Diana’s adaptability and resilience became clear when treatment plans needed to be changed, such as transitioning from active interventions to comfort-focused measures. She accepted these transitions without resentment, supporting her husband and participating actively in discussions with the interdisciplinary team to identify new ways to maintain his comfort and quality of life at home, the place where he prefers to be cared for and eventually die. Her ability to find meaning and motivation in her caregiving role is seen when she creates small daily rituals, such as sharing moments of gratitude with her husband and the nursing staff.

Mrs. Diana works closely with nurses and the medical team to facilitate spiritual and emotional well-being. For example, together they arrange times for quiet reflection, music, or favorite readings, respecting her husband’s spiritual needs alongside clinical requirements.

Throughout the process, Mrs. Diana’s humanitarian conduct and interpersonal harmony are evident in the trust she builds: she thanks the staff, acknowledges each person’s contribution, and encourages open discussion of difficult topics, including end-of-life preferences.

When discussions turn to prognosis and existential questions, Mrs. Diana’s shows transcendental awareness as she helps her husband articulate his hopes and fears honestly, and cultivates an atmosphere where family, nurses, and doctors can all reflect together on what matters most, recognizing the shared humanity in these profound conversations.

In summary, Mrs. Diana’s actions consistently demonstrate the defining attributes of spiritual intelligence within an interdisciplinary, person-centered palliative care context. Her approach promotes dignity, resilience, and holistic comfort, supported and amplified by the collaborative practice of nurses and physicians.

### 4.9. Definition of the Concept

Rodgers’ method assumes that concepts are dynamic and continually changing; thus, revising the definition of spiritual intelligence at this point in time is but a summative expression of the concept’s dominant features as known, and not intended to be final.

**Figure 2:**
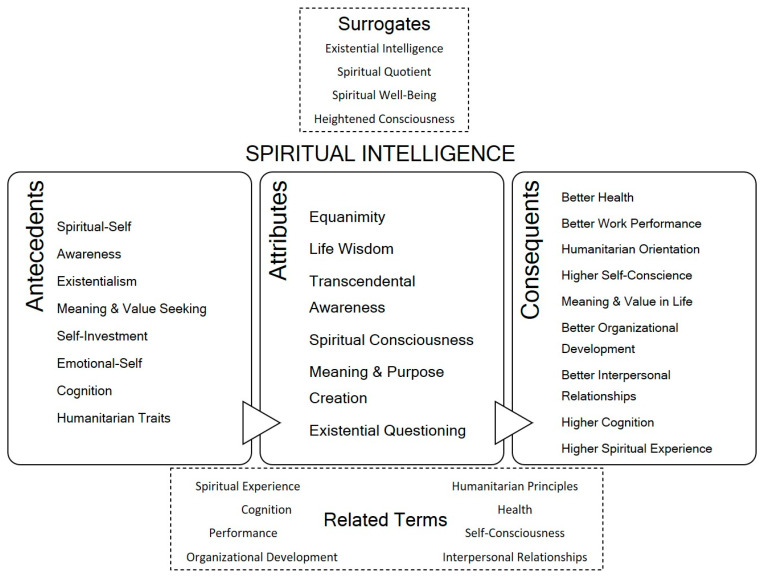
Visual diagram of spiritual intelligence concept.

Based on the attributes consistently identified across the dataset and overall characteristics repeatedly linked to those attributes (Figure 2), we defined spiritual intelligence as a distinct, unifying, and trainable intelligence characterized by the capacity to actively derive meaning, purpose, and values from lived experiences and existential questions. This intelligence involves transcendental awareness and spiritual consciousness, enabling higher cognition and the intellectual integration of spiritual insights. Furthermore, spiritual intelligence fosters equanimity, life wisdom, and a humanitarian approach by allowing individuals to effectively utilize spiritual resources for problem-solving and daily interaction, thereby transforming spirituality into action.

## 5. Discussion

Despite the effort to include multiple language literature in this analysis, the exclusion of Arabic, African and Asian native languages may limit to some extent the cultural diversity of our findings.

Nevertheless, we were able to achieve a worldwide representation of evidence through a broad time frame, which we believe is a strength in this evolutionary perspective over spiritual intelligence.

Since the concept is rather recent, we were able to gather and successfully explore literature from all fields of knowledge, giving this research a broad-spectrum approach to the concept of spiritual intelligence, allowing for a comprehensive review of its development through time.

The geographical distribution discrepancies of the studies should also be noted, as they highlight practical differences in the integration of spirituality and spiritual intelligence in daily life. Oriental cultures, where spirituality and religiosity are heavily linked to the quotidian life and even governments, show higher interest in researching the impact of spiritual intelligence and seem keener to tend to the spiritual-self. On the other hand, Occidental cultures where most governments are laic and the biomedical model originated and is still deeply rooted in many aspects of society (Hewa & Hetherington, 1995), the spiritual dimension still struggles to be seen, despite the recognition of its relevance and the recent consideration put into integrating this dimension in WHO’s health definition (Peng-Keller et al., 2022).

More than focusing on the early theoretical conceptualization of spiritual intelligence, this analysis prioritized field-research, and empirical applications drawn from it, in order to refine and update the concept in a more functional perspective.

Spiritual intelligence research was significantly amplified by the development of measuring instruments (Amram & Dryer, 2008; King & DeCicco, 2009), enabling quantitative analysis of impact and correlational investigation. This new-found evidence brought to light new outcomes in the spiritual intelligence sphere of influence and confirmed the efficiency of interventions towards spiritual intelligence enhancement (Pinto et al., 2023, 2024).

More recent studies further support the relevance and applicability of spiritual intelligence by demonstrating significant associations to moral intelligence, forgivingness and personality traits, reinforcing the construct’s links to ethical functioning, meaning-making, and personal development (Dacka & Rydz, 2023; Mróz et al., 2023). Also, in a more conceptual approach, Amram reviewed accumulated empirical and theoretical evidence, reinforcing our findings that validate spiritual intelligence based on its adaptability, cross-cultural validation, and neurological correlates, while also sharing ongoing challenges related to model convergence and measurement consistency (Amram, 2022). Although spiritual intelligence has been discussed in many contexts, no formal concept analysis has, to our knowledge, been published to date, making this review of antecedents and consequents truly insightful of the influence domain of spiritual intelligence.

Concerning antecedents, the notion that awareness, spiritual experiences, existentialism and emotional self-knowledge may precede spiritual intelligence can be major findings towards better targeted interventions (Ajele et al., 2021; Anwar et al., 2020; Augusty & Mathew, 2020; Dami et al., 2019; Imani et al., 2021; Khosravi & Nikmanesh, 2014; King et al., 2012; Parattukudi et al., 2021; Saeedi et al., 2014). Meanwhile, understanding that self-investment and seeking meaning and value are a driving force towards enhanced spiritual intelligence may help to understand how to achieve higher engagement (Abdolrezapour & Alipour, 2021; Bhandari et al., 2023; Kaur et al., 2013; Parattukudi et al., 2021; Rahmanian et al., 2018).

On the other side, consequents as positive health impact (Amirian & Fazilat-Pour, 2016; Badrudin et al., 2021; Parattukudi et al., 2021), better work performance (Martin & Hafer, 2009; Rahmawaty et al., 2021; Rani et al., 2013; Riahi et al., 2018), higher organizational development (Anwar & Gani, 2015; Dargahi & Veysi, 2021; Gera et al., 2021; Prabhu et al., 2020) and enhanced interpersonal relationships (Anwar & Gani, 2015; Geram, 2016; Mosavinezhad et al., 2019) are outcomes that can be easily measured to determine efficiency of spiritual intelligence development. These consequents also carry great policy weight since they can advocate for the benefits of investing in spiritual intelligence training of professionals in many different areas of activity, especially in those where stress and burn-out are more worrisome and when humanitarian orientation is crucial, such as healthcare, education and organizational leadership (Dargahi & Veysi, 2021; Gera et al., 2021; Hojat & Badiyepeymaiejahromi, 2021; Kaur et al., 2013; Pishghadam et al., 2022; Polemikou & Vantarakis, 2019; Prabhu et al., 2020; Rani et al., 2013; Singla et al., 2021).

Another important addition of this evolutionary concept analysis is the notion that internal consistency is fundamental to spiritual intelligence, thus, equanimity integrates the proposed definition as a core attribute, encompassing such consistency as well as a peaceful, trustful demeanor that projects from the individual to its surrounding context.

The notion that spiritual intelligent people can remain serene, wise and compassionate through challenging situations was suggested early on (Wigglesworth, 2012) but not consistently integrated in the concept definitions of different authors (King & DeCicco, 2009; Vaughan, 2002; Zohar & Marshall, 2000). This evolutionary concept analysis reinforces the claim that equanimity is a cornerstone attribute of spiritual intelligence.

Life wisdom also emerged as a novel attribute in this concept analysis. In this study, it is operationally defined as a combined psychological and spiritual capacity that grows from lived experience; it reflects the ability to navigate personal and interpersonal challenges with perseverance, discernment, and a humanitarian orientation. By integrating psychological elements (such as reflective judgment, emotional maturity, and adaptive coping), with spiritual elements (like meaning-making, ethical sensibility, and compassion), this combined capacity enables individuals to view hardship as an opportunity for growth, maintain a macro perspective in face of adversity, and respond to problems with compassion, ethical awareness, and a commitment to the wellbeing of others.

The six key attributes identified in this concept analysis: equanimity, life wisdom, transcendental awareness, spiritual consciousness, meaning and purpose creation, and existential questioning, partially overlap with constructs already assessed by existing instruments such as the SISRI-24 (King & DeCicco, 2009), the most frequently used instrument in spiritual intelligence research to date (Pinto et al., 2024). However, the newly identified attributes of equanimity and life wisdom are not represented in this instrument, highlighting a gap in this valuable measurement tool. This mismatch suggests that while SISRI-24 addresses core dimensions of spiritual intelligence, it may not fully operationalize its broader, evolving conceptualization.

This problem extends to other spiritual intelligence instruments, as for instance: Wigglesworth has somewhat reflected these new attributes in her proposed instrument while not contemplating matters of meaning and existential questioning (Wigglesworth, 2012); on another hand Amram & Dryer’s instrument, approaches some aspects of equanimity but leaves out the newly identified life wisdom attribute (Amram & Dryer, 2008).

These findings underscore the need for further development or adaptation of assessment instruments to ensure they comprehensively capture the full range of evolving spiritual intelligence attributes identified in the recent literature.

Overall, contemporary research continues to emphasize spiritual intelligence as a trainable, outcome-relevant construct, while simultaneously underscoring the need for clearer conceptual integration across models and instruments (Amram, 2022 Dacka & Rydz, 2023; Mróz et al., 2023).

## 6. Conclusions

This concept analysis demonstrates the impact of spiritual intelligence in many aspects of daily life but especially in enhancing positive health, work and interpersonal outcomes that can significantly contribute to a more resilient healthcare system.

The revised data supports an updated conceptualization of spiritual intelligence, emphasizing equanimity and life wisdom as two newly identified core attributes. These attributes expand the conceptual scope of spiritual intelligence and represent a key theoretical contribution of this work. The analysis also reinforces the importance of targeting antecedents as main focus for spiritual intelligence enhancement interventions and consequents as outcome measures to validate for such interventions.

Clarifying spiritual intelligence attributes and acknowledging its antecedents and consequents, as well as understanding how it can be successfully trained, allows for concrete policy and action towards an effective recognition and practical application of spiritual care.

Further investigation is required on the potential of targeted interventions to enhance spiritual intelligence based on this revised definition and its attributes, in order to establish its practical helpfulness. In particular, future studies should investigate whether attributes such as equanimity and life wisdom function primarily as psychological traits, spiritual dispositions, or a dynamic combination of both, and how this may change the evaluation of spiritual intelligence moving forward. Additionally, empirical studies assessing the outcomes of targeted interventions will be essential for evaluating the practical usefulness and broader implications of this refined conceptualization, along with extensive review of the existing assessment instruments to comply with the updated attributes.

## Figures and Tables

**Table 1:** Included studies on spiritual intelligence concept analysis.

Authors (Year)	Country	Setting	Participants	Study Design	Main Findings (Statistically Significant)
Abazari et al. (2025)	Iran	Healthcare	Nursing Students	Quantitative	No significant changes were found in SI with intervention (not directed at SI)
Afrashteh et al. (2025)	Iran	Community	Older Adults	Quantitative	Negative correlation between SI and death anxiety
Dávila-Valencia et al. (2025)	Peru	Healthcare	Nurses	Quantitative	Positive correlation between SI and Work Life Quality and with servant leadership.
Ghonchehpour et al. (2025)	Iran	Healthcare	Nursing and Midwifery Students	Quantitative	Positive correlation between SI and spiritual sensitivity
Grasmane et al. (2025)	Latvia	Education	Children (Primary School)	Quantitative	SI increased with targeted intervention in experimental group. Experimental group outperformed control group in post-test happiness, and spiritual well-being
Hanefar et al. (2025)	Malaysia	Community	Not Applicable	Bibliometric Analysis	Identifies 6 data clusters: mental health, well-being, leadership, workplace dynamics and the integration of SI with artificial intelligence
Kadhim et al. (2025)	Iran	Healthcare	Nurses	Quantitative	Positive correlation between SI and nurses’ moral development.
Korkut and Çetin (2025)	Turkey	Healthcare	Nursing Students	Quantitative	Positive correlation between SI and nursing professional values
Moon & Woo (2025)	Korea	Community	Older Adults	Quantitative	SI negatively mediates the link between perceived burdensomeness and suicidal ideation
Baharodin (2025)	India	Community	Not Applicable	Literature Review	Identifies 4 core themes relating SI to marital satisfaction: stability; wisdom; love; and security
Özçalık and Ataş (2025)	Turkey	Healthcare	Patients (Pregnant Women)	Quantitative	No correlations established
Fidelis et al. (2024)	Portugal	Community	Not Applicable	Literature Review	Approaches SI concept from four different perspectives (Western, Eastern, Islamic, and Hindu).
Moshashaei et al. (2024)	Iran	Healthcare	Families of Mental Health Patients	Quantitative	Positive correlation between SI and spiritual well-being based on attachment to God with mediating role of belief in a just-world, persistence in long-term goals, and self-compassion
Pinto et al. (2024)	Portugal	Community	Not Applicable	Literature Review	Outlines SI positive correlations with: resilience; general, mental, and spiritual health; emotional intelligence; and favorable social behaviors and communication strategies; and negative correlations with: burnout and stress; depression and anxiety
Shahcheragh et al. (2024)	Iran	Healthcare	Nurses	Quantitative	Negative correlation between SI and moral distress and fatigue from caregiving.
Stiliya et al. (2024)	India	Healthcare	Not Applicable	Bibliometric Analysis	Identifies increasing interest in SI within nursing research.
Walter et al. (2024)	India & Israel	University	University Students	Quantitative	Positive correlation between SI and satisfaction with life
Yüksel et al. (2024)	Turkey	Sports	Football Players	Quantitative	SI dimensions vary with marital status, age, occupation, economic income and practicing sports.
Zeladita-Huaman et al. (2024)	Peru	Healthcare	Nurses	Quantitative	Positive correlation between SI and religious practices and professional experience.
Zhou et al. (2024)	China	Education	Not Applicable	Literature Review with Meta-Analysis	States SI as an independent predictive factors in educational achievement
Amiri et al. (2023)	Iran	Healthcare	Nurses	Quantitative	Positive correlation between SI and caring behavior.
Bhandari et al. (2023)	India	Sports	Hindu Ascetics	Quantitative	Negative correlation between SI and psychological distress
Cai et al. (2023)	Pakistan	University	University Students	Quantitative	SI positively moderates the relationship between green mindfulness and green entrepreneurial intention
Dacka and Rydz (2023)	Poland	Community	Young Adults	Quantitative	Positive correlation between SI and moral intelligence
Fidelis et al. (2023)	Brasil & Portugal	Healthcare	Healthcare Workers	Quantitative	Scale validation (adapted SISRI to leader’s spiritual intelligence)
Khosravi (2023)	Iran	Healthcare	Medical Students	Quantitative	Negative correlation between SI and attitude toward euthanasia. SI negatively moderates the relationship between openness to experience and attitude toward euthanasia
Maghool et al. (2023)	Iran	Healthcare	Older Adults	Quantitative	Positive correlation between SI and fear and anxiety of corona.
Mehralian et al. (2023)	Iran	Healthcare	Nurses	Quantitative	Positive correlation between SI and competence and communication self-efficacy
Noroozi and Mohebbi-Dehnavi (2023)	Iran	Healthcare	Women with Breast Cancer	Quantitative	Positive correlation between SI and general health.
Pinto et al. (2023)	Portugal	Community	Not Applicable	Literature Review	Lists covered topics in SI interventions: self-awareness; self-management; self-consciousness; meaning in life; sense of holiness and interpersonal relationships
Rafiei et al. (2023)	Iran	Healthcare	Patients (Diabetics	Quantitative	SI predicts 7.2% of change in self-management among diabetes patients
Rajabi et al. (2023)	Iran	Healthcare	Drugs Users	Quantitative	SI was higher in the group of narcotics anonymous (counseling only) than that in the methadone group
Senmar et al. (2023)	Iran	University	University Students (Medical Sciences)	Quantitative	Positive correlation between SI and life satisfaction.
Yadollahpour et al. (2023)	Iran	Healthcare	Nurses	Quantitative	Negative correlation between SI and occupational stress
Zamani et al. (2023)	Iran	Healthcare	Medical Students	Quantitative	Negative correlation between SI and occupational stress
Zolfaghary et al. (2023)	Iran	Healthcare	Midwives	Quantitative	Negative correlation between SI and stress
Grasmane et al. (2022)	Latvia	Education	Children (Primary School)	Quantitative	Children scale validation
Marzban et al. (2022)	Iran	Healthcare	Family Caregivers of Mental Health Patients	Quantitative	Positive correlation between SI and resilience
Mokhtari et al. (2022)	Iran	Healthcare	Infertile Couples	Quantitative	No correlations established
Pishghadam et al. (2022)	Iran	Education	English as a Foreign Language Teachers	Quantitative	Negative correlation between SI and psychological reactance and burnout
Sharifnia et al. (2022a)	Iran	Healthcare	Not Applicable	Literature Review with Meta-Analysis	Positive correlation between SI and professional nursing practice components: the art of nursing; competence; attributes of practice; and personal commitment
Sharifnia et al. (2022b)	Iran	Healthcare	Not Applicable	Literature Review with Meta-Analysis	Enumerates the following benefits from SI interventions: increased SI; better communication skills; job satisfaction; spiritual care competence; and decrease in overall stress.
Abdolrezapour and Alipour (2021)	Iran	Education	English as a Foreign Language Female Students	Mixed-Methods	SI increased with targeted intervention in experimental group. Experimental group outperformed control group in post-test willingness to communicate.
Ahmadi et al. (2021)	Iran	University	University Students (Nursing)	Quantitative	Positive correlation between SI and spiritual care competence
Ajele et al. (2021)	Nigeria	Healthcare	Patients (Diabetics)	Quantitative	Positive correlation between SI and mental well-being and mindfulness. Negative correlation between SI and emotional dysregulation
Alamanda et al. (2021)	Malaysia	Organizations	Employees (Manufacturing & Service)	Quantitative	Positive correlation between SI and organizational citizenship behavior
Aliabadi et al. (2021)	Iran	Healthcare	Nurses	Quantitative	Positive correlation between SI and empathy
Atroszko et al. (2021)	Poland	Community	General Population	Quantitative	Scale validation (unfit model)
Badrudin et al. (2021)	Malaysia	Healthcare	University Students (Healthcare)	Quantitative	Positive correlation between SI and spiritual health
Dargahi & Veysi (2021)	Iran	University	Employees (Academic & Administrative)	Quantitative	Positive correlation between SI and employees’ organizational commitment
Gera et al. (2021)	India	University	University Students (Finance)	Mixed-Methods	Positive correlation between SI dimensions and academic performance
Hojat and Badiyepeymaiejahromi (2021)	Iran	Healthcare	Nurses	Quantitative	Positive correlation between SI and professional self-concept
Imani et al. (2021)	Iran	Healthcare	University Students (Healthcare)	Quantitative	Positive correlation between SI and clinical competency
Liu et al. (2021)	China	Education	Primary School Teachers	Quantitative	SI varies with professional title, working location and age. Positive correlation between SI and awe. SI positively mediates relationship between awe and life satisfaction
Mróz et al. (2021)	Poland	Community	General Population	Quantitative	Positive correlation between SI and forgivingness
Oyewunmi et al. (2021)	Nigeria	University	Employees (Academic & Administrative)	Quantitative	Positive correlation between SI and job performance, job commitment and job satisfaction
Özsarı and Ilkim (2021)	Turkey	Sports	Physically Handicapped Badminton Players	Quantitative	SI dimensions vary with gender, age, marital status and sports experience
Parattukudi et al. (2021)	Canada	Healthcare	Patients (Mental Health)	Quantitative	Negative correlation between SI and depression
Rahmawaty et al. (2021)	Indonesia	Organizations	Employees (Microfinance)	Quantitative	Positive correlation between SI and communication competence and employee performance
Singla et al. (2021)	England	Education	Teachers	Quantitative	Positive correlation between SI and quality of work life
Anwar et al. (2020)	Malaysia	University	University Students (Finance)	Quantitative	Positive correlation between SI and emotional intelligence
Arnout (2020)	Egypt	Community	Unemployed People	Quantitative	SI negatively mediates the relationship between unemployed stress and mental health
Arsang-Jang et al. (2020)	Iran	Healthcare	Nurses	Quantitative	Positive correlation between SI and higher lever ethical decision making
Augusty and Mathew (2020)	India	Community	Not Applicable	Literature Review with Meta-Analysis	Proposes that SI is essential for holistic growth in developing nations
Martín-Sánchez et al. (2020)	Spain	Community	General Population	Mixed-Methods	Scale validation. Positive correlation between SI and resilience
Prabhu et al. (2020)	India	University	Not Applicable	Literature Review	Defines a new conceptual model of interaction between SI and spiritual leadership
Vasconcelos (2020)	Brazil	Community	Not Applicable	Literature Review	Proposes SI as the way to achieve and work on personal spirituality.
Dami et al. (2019)	Indonesia	University	University Students	Quantitative	SI increased with targeted intervention in experimental group. Experimental group post-test depression, anxiety and stress decreased significantly more than for control group.
Ebrahimi et al. (2019)	Iran	Healthcare	Rehabilitation Experts	Quantitative	Positive correlation between SI and resilience
Feng et al. (2019)	China	Community	Students, Employees & General Population	Mixed-Methods	Scale validation
Ghalaychi et al. (2019)	Iran	Healthcare	Midwives & Patients	Quantitative	Positive correlation between SI and patients’ satisfaction
Hatami et al. (2019)	Iran	Healthcare	Patients (Pregnant Women)	Quantitative	Positive correlation between SI and resilience
Mosavinezhad et al. (2019)	Iran	University	University Students (Psychology)	Quantitative	Negative correlation between SI and social anxiety
Sharif et al. (2019)	Iran	Organizations (Military)	Disabled Veterans	Quantitative	Positive correlation between SI and death anxiety
Polemikou et al. (2019)	Greece	Community	General Population	Quantitative	Scale validation. Positive correlation between SI and resilience and meaning in life
Polemikou and Vantarakis (2019)	Greece	Healthcare	Emergency First Responders	Quantitative	Positive correlation between SI and death anxiety and dissociative experiences
Rahmanian et al. (2019)	Iran	Healthcare	Patients (Adolescents with Type 1 Diabetes)	Quantitative	No correlations established
Sharma (2019)	India	Community	Professional & Non-professional Working Women	Quantitative	Positive correlation between SI and adjustment
Abdollahpour and Khosravi (2018)	Iran	Healthcare	Patients (Pregnant Women)	Quantitative	Positive correlation between SI and happiness. Negative correlation between SI and fear of childbirth
Antunes et al. (2018)	Portugal	Community	General Population	Quantitative	Scale validation. Positive correlation between SI and meaning and purpose at work
Baloochi et al. (2018)	Iran	University	University Students (Healthcare)	Quantitative	SI varies with age, marital status and academic background. Negative correlation between SI and aggression
Fazlolah (2018)	Iran	Education	High School Teachers	Quantitative	Positive correlation between SI and informative, normative and commitment identity styles
Mahmood et al. (2018)	Malaysia	Organizations/Human Resources	Not Applicable	Literature Review	Suggests that SI is key to organizational development through conceptual connections to human resources and leadership development, holistic mechanisms and sustainability
Malini and Raju (2018)	India	University	University Students	Quantitative	Positive correlation between SI and motivation
Rahmanian et al. (2018)	Iran	Healthcare	Patients	Quantitative	Positive correlation between SI and self-efficacy
Riahi et al. (2018)	Iran	Healthcare	Nurses	Quantitative	SI and spiritual care competence increased with targeted intervention in experimental group. Experimental group outperformed control group in post-test spiritual care competence
Zarrinabadi et al. (2018)	Iran	University	Univesity Students (Medical Librarianship)	Quantitative	Confirmed high internal correlation among SI dimensions
Udin et al. (2017)	Indonesia	Organizations	Engineers	Quantitative	Positive correlation between SI and affective commitment and job performance
Amirian and Fazilat-Pour (2016)	Iran	University	University Students	Quantitative	Positive correlation between SI and general health and happiness
Chan and Siu (2016)	China	University	University Students	Quantitative	Scale validation. Positive correlation between SI and meaning in life and metapersonal self-construal
Geram (2016)	Iran	Education	Male Teachers	Quantitative	Positive correlation between SI and social adjustment
Jorge et al. (2016)	Portugal	Community	General Population	Quantitative	Scale validation
Anwar and Gani (2015)	Malaysia	Organizations	Employees (Manufacturing & Service)	Quantitative	Positive correlation between SI and organizational citizenship behavior
Mahasneh et al. (2015)	Jordan	University	University Students	Quantitative	Positive correlation between SI dimensions and personality traits
Khosravi and Nikmanesh (2014)	Iran	University	University Students	Quantitative	Positive correlation between SI and resilience. Negative correlation between SI and perceived stress
Nekouei et al. (2014)	Iran	Healthcare	Patients (Coronary Heart Disease)	Quantitative	SI is the main psychological protective factor for coronary heart disease
Saeedi et al. (2014)	Iran	University	Female University Students	Quantitative	Positive correlation between SI and religious orientation
Azizi and Zamaniyan (2013)	Iran	University	English as a Foreign Language Students	Quantitative	Positive correlation between SI and metacognitive and social strategies
Azizollah et al. (2013)	Iran	University	University Students	Quantitative	Positive correlation between SI and emotional intelligence and academic achievement
Hassan and Shabani (2013)	Iran	Education	Adolescents	Quantitative	Negative correlation between SI and mental health problems
Kaur et al. (2013)	Malaysia	Healthcare	Nurses and Patients	Quantitative	Positive correlation between SI and emotional intelligence and psychological ownership towards job. The relationship between SI and caring behavior is positively mediated by professional ownership and negatively mediated by burnout
Rani et al. (2013)	Malaysia	Healthcare	Nurses	Quantitative	Positive correlation between SI and work performance
Shahbakhsh and Moallemi (2013)	Iran	Healthcare	Patients (Addicts)	Quantitative	Positive correlation between SI and drug withdrawal time and resilience
Babanazari et al. (2012)	Iran	Education	Adolescents	Quantitative	Positive correlation between SI and happiness
King et al. (2012)	Canada	University	University Students & General Population	Quantitative	Positive correlation between SI and emotional intelligence
Narayanan and Jose (2011)	India	Education/Religion	Adolescents	Quantitative	Positive correlation between SI dimensions and resilience
King and DeCicco (2009)	Canada	University	University Students (Psychology)	Quantitative	Scale validation. Positive correlation between SI and meaning of life, metapersonal self-construal, mysticism, desirable responding and intrinsic-extrinsic religiosity
Martin and Hafer (2009)	USA	University	University Students (Finance)	Quantitative	Positive correlation between SI and emotional intelligence
Yang and Wu (2009)	China	Healthcare	Nurses	Quantitative	SI varies with academic background, social system and religious upbringing
Yang and Mao (2007)	China	Healthcare settings	Nurses	Quantitative	SI varies with religious affiliation and beliefs
Tirri et al. (2005)	Kosovo	Organizations (Military)	Peacekeepers	Quantitative	Scale validation

Footnote: SI, spiritual intelligence. Full article reference details available in Supplementary Material: Table S1.

## Data Availability

The raw data supporting the conclusions of this article will be made available by the authors on request.

## Supplementary Materials

The following supporting information can be downloaded at: https://www.mdpi.com/article/10.3390/jintelligence14020024/s1, Table S1: Included studies on spiritual intelligence concept analysis full reference details. Table S2: Spiritual Intelligence areas of application analysis. Table S3: Spiritual intelligence surrogate terms analysis. Table S4: Spiritual intelligence-related terms analysis. Table S5: Spiritual intelligence antecedents’ analysis. Table S6: Spiritual intelligence consequents’ analysis. Table S7: Spiritual intelligence attributes’ analysis.

## Abbreviations

The following abbreviations are used in this manuscript:

PRISMA-ScR	PRISMA extension for scoping reviews
AI	Artificial Intelligence
