# Supplementary material for: Spiritual Intelligence: A Scoping Review with Concept Analysis on the Key to Spiritual Care
Source: J Intell. 2026 Feb 3;14(2):24. doi: 10.3390/jintelligence14020024 (PMC12942164; doi:10.3390/jintelligence14020024)
Supplement: Supplementary file 1 [file jintelligence-14-00024-s001.zip › jintelligence-3978296-supplementary.pdf]

Supplementary Materials

Table S1. Included studies on spiritual intelligence concept analysis full reference details.

**Author(s). (Year). Title of the article. *Title of the Journal*, volume number (issue number), page range. https://doi.org/xxxx**
(Abazari et al., 2025) Abazari, L., Abazari, A., Emamgholi, M., & Asadi, N. (2025). Effect of Pranayama Practice on Emotional Intelligence and Spiritual Intelligence in Nursing Students. SAGE Open Nursing, 11. https://doi.org/10.1177/23779608251371103
(Afrashteh et al., 2025) Afrashteh, M. Y., Fartash, A., & Aboutorabi, K. (2025). Relationship between death anxiety and spiritual intelligence: the potential mediating role of meaning of life and resilience in Iranian older people. BMC Geriatrics, 25(1). https://doi.org/10.1186/s12877-025-06040-2
(Dávila-Valencia et al., 2025) Dávila-Valencia, P. K., Gala-Espinoza, B. J., & Morales-García, W. C. (2025). Spiritual Intelligence in Healthcare Practice and Servant Leadership as Predictors of Work Life Quality in Peruvian Nurses. Nursing Reports, 15(7). https://doi.org/10.3390/nursrep15070249
(Ghonchehpour et al., 2025) Ghonchehpour, A., Afshar, P. P., Tirgari, B., Maazallahi, M., & Forouzi, M. A. (2025). Spiritual sensitivity in nursing and midwifery students and its relationship with spiritual intelligence and spiritual growth. BMC Medical Education, 25(1). https://doi.org/10.1186/s12909-025-08041-6
(Grasmane et al., 2025) Grasmane, I., Pipere, A., & Raščevskis, V. (2025). Effectiveness of a Psycho-pedagogical Intervention on Spiritual Intelligence, Happiness, and Spiritual Well-being for Primary School Children: A Non-randomized Controlled Trial. Journal of Happiness Studies, 26(1). https://doi.org/10.1007/s10902-024-00844-6
(Hanefar et al., 2025) Hanefar, S. B. M., Benaouda, B., Faizuddin, A., & Ramachandaran, S. D. (2025). Mapping the Landscape of Spiritual Intelligence: A Bibliometric Analysis of Trends, Patterns and Future Directions. Journal of Religion and Health, 64(5), 3419–3447. https://doi.org/10.1007/s10943-025-02386-4
(Kadhim et al., 2025) Kadhim, A. J., Kadhim, M. H., Noghabi, E. S., Taher, M., Barzgar, B., Masabi, N. S., Eftekhari, A., Chenari, H. A., & Baumann, S. L. (2025). Exploring the Relationship Between Nurses' Spiritual Intelligence, Critical Thinking, and Moral Development in the Middle East. Nursing Science Quarterly. https://doi.org/10.1177/08943184251388292
(Korkut & Çetin, 2025) Korkut, S., & Çetin, B. (2025). The Relationship Between Spiritual Intelligence and Compliance with Professional Values in Nursing Students in Türkiye. Journal of Religion and Health, 64(3), 1770–1782. https://doi.org/10.1007/s10943-025-02291-w

(Moon & Woo, 2025) Moon, A., & Woo, S. (2025). Perceived burdensomeness, spiritual intelligence, and suicidal ideation: Implications for attitudes toward physician-assisted suicide in Korean Older Adults. Journal of Health Psychology. https://doi.org/10.1177/13591053251369358
(Normarina & Baharodin, 2025) Normarina, Misuari, & Baharodin. (2025). A Systematic Literature Review on The Importance of Spiritual Intelligence in Marital Satisfaction. Akademika, 95(03). https://doi.org/10.17576/akad-2025-9503-12
(Özçalık & Atas, 2025) Özçalık, H. B., & Ataş, A. N. (2025). The Effects of Prenatal Optimism and Spiritual Intelligence Levels on Childbirth Attitudes. Clinical and Experimental Health Sciences, 15(1), 35–41. https://doi.org/10.33808/clinexphealthsci.1362979
(Fidelis et al., 2024) Fidelis, A., Moreira, A. C., & Vitória, A. (2024). Multiple perspectives of spiritual intelligence: A systematic literature review. Social Sciences and Humanities Open, 9. https://doi.org/10.1016/j.ssaho.2024.100879
(Moshashaei et al., 2024) Moshashaei, S. K. D., Yarahmadi, Y., & Sharifi, H. P. (2024). The causal model of spiritual well-being based on an accompanist of god and spiritual intelligence. Journal of Education and Health Promotion, 13(1). https://doi.org/10.4103/jehp.jehp_1283_23
(Pinto et al., 2024) Pinto, C. T., Guedes, L., Pinto, S., & Nunes, R. (2024). Spiritual intelligence: a scoping review on the gateway to mental health. Global Health Action, 17(1). https://doi.org/10.1080/16549716.2024.2362310
(Shahcheragh et al., 2024) Shahcheragh, S. H., Fekri, N., & Rad, M. (2024). The Relationship between Spiritual Intelligence and Fatigue and Moral Distress in Emergency Nurses: A Cross-Sectional Study. Iranian Journal of Nursing and Midwifery Research, 29(6), 737–742. https://doi.org/10.4103/ijnmr.ijnmr_157_23
(Stiliya et al., 2024) Stiliya, J. K., Antony, J. M., & Joseph, J. (2024). Spiritual Intelligence and Spiritual Care in Nursing Practice: A Bibliometric Review. Indian Journal of Palliative Care, 30(4), 304–314. https://doi.org/10.25259/IJPC_155_2024
(Walter et al., 2024) Walter, O., Kasler, J., & Routray, S. (2024). Emotional intelligence, spiritual intelligence, depression and anxiety, and satisfaction with life among emerging adults in Israel and India: the impact of gender and individualism/collectivism. BMC Psychology, 12(1). https://doi.org/10.1186/s40359-024-01806-6
(Yüksel et al., 2024) Yüksel, O., Addeen Ra'ed Alkafaween, S., Demir, O., Oruç, A., Erdem, N., Shamatava, K., Çakto, P., & Karakullukçu, A. (2024). Investigation of spiritual intelligence levels of active football players. Retos, 58, 796–803. https://recyt.fecyt.es/index.php/retos/index

(Zeladita-Huaman et al., 2024) Zeladita-Huaman, J. A., Cuba-Sancho, J. M., Martina-Chávez, M. B., Zegarra-Chapoñan, R., & Castillo-Parra, H. (2024). Religion and professional experience: Are they predictors of nurses' spiritual intelligence? Cross-sectional study. Revista Brasileira de Enfermagem, 77(6). https://doi.org/10.1590/0034-7167-2024-0217
(Zhou et al., 2024) Zhou, Z., Tavan, H., Kavarizadeh, F., Sarokhani, M., & Sayehmiri, K. (2024). The relationship between emotional intelligence, spiritual intelligence, and student achievement: a systematic review and meta-analysis. BMC Medical Education, 24(1). https://doi.org/10.1186/s12909-024-05208-5
(Amiri et al., 2023) Amiri, R., Gaeeni, M., & Tehran, H. A. (2023). The mediating role of moral reasoning in spiritual intelligence and caring behaviors in Iranian emergency nurses. J. Med. Ethics. Hist. Med, 16(11).
(Bhandari et al., 2023) Bhandari, R. B., Chaudhry, N., & Devi, S. (2023). Relationship Between Spirituality and Distress in Ascetics. *Psychological Reports*, 126(1), 169–180. https://doi.org/10.1177/00332941211043454
(Cai et al., 2023)Cai, B., Chen, Y., & Ayub, A. (2023). "Quiet the Mind, and the Soul Will Speak"! Exploring the Boundary Effects of Green Mindfulness and Spiritual Intelligence on University Students' Green Entrepreneurial Intention–Behavior Link. Sustainability (Switzerland), 15(5). https://doi.org/10.3390/su15053895
(Dacka & Rydz, 2023) Dacka, M., & Rydz, E. (2023). Personality Traits and the Spiritual and Moral Intelligence of Early Adulthood in Poland: Research Reports. Religions, 14(1). https://doi.org/10.3390/rel14010078
(Fidelis et al., 2023) Fidelis, A. C. F., Formiga, N. S., & Fernandes, A. J. (2023). A measure of leadership's spiritual intelligence from the perspectives of Brazilian and Portuguese workers. Revista CES Psicologia, 16(2), 17–30. https://doi.org/10.21615/cesp.6405
(Khosravi, 2023) Khosravi, M. (2023). The impact of openness to experience personality trait on attitudes of medical students toward euthanasia: the moderating role of spiritual intelligence. European Journal of Translational Myology, 33(4). https://doi.org/10.4081/ejtm.2023.11845
(Maghool et al., 2023) Maghool, A., Bakhshi, M., Rastaghi, S., & Rad, M. (2023). Relationship between spiritual intelligence and intolerance of uncertainty, anxiety and fear of Corona in the elderly. Journal of Education and Health Promotion, 12(1), 399. https://doi.org/10.4103/jehp.jehp_1623_22
(Mehralian et al., 2023) Mehralian, G., Yusefi, A. R., Dastyar, N., & Bordbar, S. (2023). Communication competence, self-efficacy, and spiritual intelligence: evidence from nurses. BMC Nursing, 22(1). https://doi.org/10.1186/s12912-023-01262-4

(Noroozi & Mohebbi-Dehnavi, 2023) Noroozi, M., & Mohebbi-Dehnavi, Z. (2023). Investigating the relationship between religious orientation and spiritual intelligence with general health dimensions in women with breast cancer. Journal of Education and Health Promotion, 12(1), 40. https://doi.org/10.4103/jehp.jehp_1744_21
(Pinto et al., 2023) Pinto, C. T., Veiga, F., Guedes, L., Pinto, S., & Nunes, R. (2023). Models of spiritual intelligence interventions: A scoping review. Nurse Education in Practice, 73, 103829. https://doi.org/10.1016/j.nepr.2023.103829
(Rafiei et al., 2023) Rafiei, S., Souri, S., Nejatifar, Z., & Amerzadeh, M. (2023). The relationship between spiritual intelligence and self-management in patients with diabetes type 1. BMC Endocrine Disorders, 23(1). https://doi.org/10.1186/s12902-023-01482-4
(Rajabi et al., 2023) Rajabi, R., Aliabadi, H. E., Mahdizadeh, M. J., & Forouzi, M. A. (2023). A comparative study of religious beliefs, spiritual intelligence and spiritual well-being in two therapies based on education (anonymous drug user) and methadone in drug user in Iran. BMC Research Notes, 16(1). https://doi.org/10.1186/s13104-023-06377-0
(Senmar et al., 2023) Senmar, M., Azimian, J., Noorian, S., Aliakbari, M., & Chegini, N. (2023). Relationship between spiritual intelligence and lifestyle with life satisfaction among students of medical sciences. BMC Medical Education, 23(1). https://doi.org/10.1186/s12909-023-04506-8
(Yadollahpour et al., 2023) Yadollahpour, M. H., Nouriani, M., Faramarzi, M., Yaminfirooz, M., Shams, M. A., & Gholinia, H. (2023). Role of spiritual intelligence and demographic factors as predictors of occupational stress, quality of life and coronavirus anxiety among nurses during the COVID-19 pandemic. Nursing Open, 10(3), 1449–1460. https://doi.org/10.1002/nop2.1395
(Zamani et al., 2023) Zamani, A., Ghaffari, M., Mohseny, M., & Rakhshanderou, S. (2023). The Effect of Spiritual Intelligence on Occupational Stress Among Medical Interns During COVID-19 Pandemic: A Structural Equation Model. Iranian Journal of Psychiatry and Behavioral Sciences, 17(3). https://doi.org/10.5812/ijpbs-131368
(Zolfaghary et al., 2023) Zolfaghary, F., Osko, S., Bakouei, F., Pasha, H., & Adib-Rad, H. (2023). Spiritual Intelligence as a Coping Strategy to Manage Job Stress for Midwives in Northern Iran: A Cross-Sectional Study. Journal of Religion and Health, 62(5), 3301–3312. https://doi.org/10.1007/s10943-023-01863-y
(Grasmane et al., 2022) Grasmane, I., Raščevskis, V., & Pipere, A. (2022). Primary validation of Children Spiritual Intelligence Scale in a sample of Latvian elementary school pupils. International Journal of Children’s Spirituality, 27(2), 97–112. https://doi.org/10.1080/1364436X.2022.2043833

(Marzban et al., 2022) Marzban, A., Fereidooni-Moghadam, M., & Ghezelbash, S. (2022). The relationship between spiritual intelligence and resilience in family caregivers of patients with chronic mental disorders. Perspectives in Psychiatric Care, 58(4), 2846–2853. https://doi.org/10.1111/ppc.13132
(Mokhtari et al., 2022) Mokhtari, F., Torabi, F., & Pirhadi, M. (2022). Relationship between fertility characteristics with spiritual intelligence and resilience in infertile couples. Journal of Education and Health Promotion, 11(1). https://doi.org/10.4103/jehp.jehp_97_21
(Pishghadam et al., 2022) Pishghadam, R., Yousofi, N., Amini, A., & Sadat Tabatabayeeyan, M. (2022). Interplay of psychological reactance, burnout, and spiritual intelligence: A case of Iranian EFL teachers. *Revista de Psicodidactica*, 27(1), 76–85. https://doi.org/10.1016/j.psicod.2021.06.001
(Sharifnia et al., 2022a) Sharifnia, A. M., Fernandez, R., Green, H., & Alananzeh, I. (2022a). Spiritual intelligence and professional nursing practice: A systematic review and meta-analysis. International Journal of Nursing Studies Advances, 4. https://doi.org/10.1016/j.ijnsa.2022.100096
(Sharifnia et al., 2022b) Sharifnia, A. M., Fernandez, R., Green, H., & Alananzeh, I. (2022b). The effectiveness of spiritual intelligence educational interventions for nurses and nursing students: A systematic review and meta-analysis. Nurse Education in Practice, 63. https://doi.org/10.1016/j.nepr.2022.103380
(Abdolrezapour & Alipour, 2021) Abdolrezapour, P., & Alipour, J. (2021). Fostering spiritual intelligence and a concomitant development of WTC in EFL learners. *Teaching Theology and Religion*, 24(3), 136–151. https://doi.org/10.1111/teth.12592
(Ahmadi et al., 2021) Ahmadi, M., Estebsari, F., Poormansouri, S., Jahani, S., & Sedighie, L. (2021). Perceived professional competence in spiritual care and predictive role of spiritual intelligence in Iranian nursing students. *Nurse Education in Practice*, 57. https://doi.org/10.1016/j.nepr.2021.103227
(Ajele et al., 2021) Ajele, W. K., Oladejo, T. A., Akanni, A. A., & Babalola, O. B. (2021). Spiritual intelligence, mindfulness, emotional dysregulation, depression relationship with mental well-being among persons with diabetes during COVID-19 pandemic. *Journal of Diabetes and Metabolic Disorders*, 20(2), 1705–1714. https://doi.org/10.1007/s40200-021-00927-8
(Alamanda et al., 2021) Alamanda, D. T., Ahmad, I., Putra, H. D., & Hashim, N. A. (2021). The role of spiritual intelligence in citizenship behaviours amongst muslim staff in Malaysia. *HTS Teologiese Studies / Theological Studies*, 77(1). https://doi.org/10.4102/hts.v77i1.6586
(Aliabadi et al., 2021) Aliabadi, P. K., Zazoly, A. Z., Sohrab, M., Neyestani, F., Nazari, N., Mousavi, S. H., Fallah, A., Youneszadeh, M., Ghasemiyan, M., & Ferdowsi, M. (2021). The role of spiritual intelligence in predicting the empathy levels of nurses with COVID-19 patients. *Archives of Psychiatric Nursing*, 35(6), 658–663. https://doi.org/10.1016/j.apnu.2021.10.007

(Atroszko et al, 2021) Atroszko, P. A., Skrzypińska, K., & Balcerowska, J. M. (2021). Is There a General Factor of Spiritual Intelligence? Factorial Validity of the Polish Adaptation of Spiritual Intelligence Self-Report Inventory. *Journal of Religion and Health*, 60(5), 3591–3605. https://doi.org/10.1007/s10943-021-01350-2
(Badrudin et al., 2021) Badrudin, Komariah, A., Wijaya, C., Barowi, Akib & Samrin (2021). Effect of spiritual intelligence on spiritual health during the COVID-19 pandemic. *HTS Teologiese Studies/Theological Studies*. https://doi.org/10.4102/hts.v77i1.6594
(Dargahi & Veysi, 2021) Dargahi, H., & Veysi, F. (2021). The relationship between managers' ideal intelligence as a hybrid model and employees' organizational commitment: a case study in Tehran University of Medical Sciences. *In Journal of Medical Ethics and History of Medicine* (Vol. 14, Issue 8). https://doi.org/10.18502/jmehm.v14i8.6752
(Gera et al., 2021) Gera, N., Vesperi, W., Di Fatta, D., Sahni, A., & Arora, A. (2021). Human resource development and spiritual intelligence: an investigation amongst management students in Delhi NCR. *International Journal of Innovation and Learning*, 29(1). https://doi.org/10.1504/IJIL.2021.111831
(Hojat & Badiyepeymaiejahromi, 2021) Hojat, M., & Badiyepeymaiejahromi, Z. (2021). Relationship between Spiritual Intelligence and Professional Self-concept among Iranian Nurses. *Investigacion y Educacion En Enfermeria*, 39(3). https://doi.org/10.17533/udea.iee.v39n3e12
(Imani et al., 2021) Imani, B., Imani, G., & Karampourian, A. (2021). Correlation between Spiritual Intelligence and Clinical Competency in Students Who Are Children of War Victims. *Iranian Journal of Psychiatry* 2021; 16(3), 329-335.
(Liu et al., 2021) Liu, Z., Li, X., Jin, T., Xiao, Q., & Wuyun, T. (2021). Effects of Ethnicity and Spiritual Intelligence in the Relationship Between Awe and Life Satisfaction Among Chinese Primary School Teachers. *Frontiers in Psychology*, 12. https://doi.org/10.3389/fpsyg.2021.673832
(Mróz et al., 2021) Mróz, J., Kaleta, K., & Skrzypińska, K. (2021). The role of spiritual intelligence and dispositional forgiveness in predicting episodic forgiveness. *Journal of Beliefs and Values*, 42(4), 415–435. https://doi.org/10.1080/13617672.2020.1851555
(Oyewunmi et al., 2021) Oyewunmi, A. E., Esho, E., & Oyewunmi, O. A. (2021). Spiritual intelligence and employee outcomes in an African sample. *Journal of Management, Spirituality & Religion*, 18(2), 151–171. https://doi.org/10.51327/ZPDN7247
(Özsarı & Ilkim , 2021) Özsarı, A., & Ilkim, M. (2021). Investigation of the Spiritual Intelligence Features of Physically Handicapped Badminton Players in Terms of Various Variables. *International Journal of Life Science and Pharma Research*. https://doi.org/10.22376/ijpbs/ijlpr/SP14/jan/2021.1-295

(Parattukudi et al., 2021) Parattukudi, A., Maxwell, H., Dubois, S., & Bédard, M. (2021). Women's Spiritual Intelligence is Associated With Fewer Depression Symptoms: Exploratory Results From a Canadian Sample. *Journal of Religion and Health*, 61(1), 433–442. https://doi.org/10.1007/s10943-021-01412-5
(Rahmawaty et al., 2021) Rahmawaty, A., Rokhman, W., Bawono, A., & Irkhami, N. (2021). Emotional intelligence, spiritual intelligence and employee performance: The mediating role of communication competence. *International Journal of Business and Society*, 22(2), 734–752. https://doi.org/10.33736/ijbs.3754.2021
(Singla et al., 2021) Singla, H., Mehta, M. D., & Mehta, P. (2021). Modeling spiritual intelligence on quality of work life of college teachers: a mediating role of psychological capital. *International Journal of Quality and Service Sciences*, 13(3), 341–358. https://doi.org/10.1108/IJQSS-07-2020-0108
(Anwar et al., 2020) Anwar, A., Gani, A. M. O., & Rahman, M. S. (2020). Effects of spiritual intelligence from Islamic perspective on emotional intelligence. *Journal of Islamic Accounting and Business Research*, 11(1), 216–232. https://doi.org/10.1108/JIABR-10-2016-0123
(Arnout, 2020) Arnout, B. A. (2020). A structural equation model relating unemployment stress, spiritual intelligence, and mental health components: Mediators of coping mechanism. *Journal of Public Affairs*, 20(2). https://doi.org/10.1002/pa.2025
(Arsang-Jang et al., 2020) Arsang-Jang, S., Khoramirad, A., Pourmarzi, D., & Raisi, M. (2020). Relationship Between Spiritual Intelligence and Ethical Decision Making in Iranian Nurses. *Journal of Humanistic Psychology*, 60(3), 330–341. https://doi.org/10.1177/0022167817704319
(Augusty & Mathew, 2020) Augusty, P. A., & Mathew, J. (2020). Spiritual intelligence and the knowledge society a systematic review of literature to understand and examine the importance of spiritual intelligence in a knowledge society. *Journal of Critical Reviews*, 7(19), 585–594. https://doi.org/10.31838/jcr.07.19.72
(Martín-Sánchez et al., 2020) Martín-Sánchez, A., Rodríguez-Zafra, M., & Ceniceros-Estévez, J. C. (2020). El estudio de la Inteligencia Espiritual II: un instrumento de evaluación de su desempeño competencial. *Acción Psicológica*, 17(2). https://doi.org/10.5944/ap.17.2.29528
(Prabhu et al., 2020) Prabhu, C. J., Mehta, M., & Srivastava, A. P. (2020). A new model of practical spiritual intelligence for the leadership development of human capital in Indian Universities. *Journal of Applied Research in Higher Education*. https://doi.org/10.1108/JARHE-11-2019-0296
(Vasconcelos, 2020) Vasconcelos, A. F. (2020). Spiritual intelligence: a theoretical synthesis and work-life potential linkages. *International Journal of Organizational Analysis*, 28(1), 109–134. https://doi.org/10.1108/IJOA-04-2019-1733

(Dami et al., 2019) Dami, Z. A., Setiawan, I., Sudarmanto, G., & Lu, Y. (2019). Effectiveness of group counseling on depression, anxiety, stress and components of spiritual intelligence in student. *International Journal of Scientific and Technology Research*, 8(9), 236–243.
(Ebrahimi et al., 2019) Ebrahimi, B., Hosseini, M., Abdi, K., Bakhshi, E., & Shirozhan, S. (2019). The Relationship between Spiritual Intelligence and Resiliency of Rehabilitation Staff. *Journal of Pastoral Care & Counseling*, 73(4), 205–210. https://doi.org/10.1177/1542305019877158
(Feng et al., 2019) Feng, M., Xiong, X. Y., & Li, J. J. (2019). Spiritual Intelligence Scale--Chinese Form: Construction and Initial Validation. *Current Psychology*, 38(5), 1318–1327. https://doi.org/10.1007/s12144-017-9678-5
(Ghalaychi et al., 2019) Ghalaychi, Z., Dolatian, M., Mahmoodi, Z., Boromandnia, N., & Valizadeh, R. (2019). The Relationship between Spiritual Health and Spiritual Intelligence of Midwives and Midwifery Students and Satisfaction of Clients with Vaginal Childbirth. *Journal of Pharmaceutical Research International*, 28(5), 1–8. https://doi.org/10.9734/jpri/2019/v28i530215
(Hatami et al., 2019) Hatami, A., Badrani, M. R., Kamboo, M. S., Jahangirimehr, A., & Hemmatipour, A. (2019). An Investigation of the Relationship of Spiritual Intelligence and Resilience With Attitude To Fear of Childbirth in Pregnant Women. *Journal of Evolution of Medical and Dental Sciences*, 8(1), 24–28. https://doi.org/10.14260/jemds/2019/6
(Mosavinezhad et al., 2019) Mosavinezhad, S. M., Safara, M., Kasir, S., & Khanbabaee, M. (2019). Role of Spiritual Intelligence and Personal Beliefs in Social Anxiety among University Students. *Health, Spirituality and Medical Ethics*, 6(3), 11–17. https://doi.org/http://dx.doi.org/10.29252/jhsme.6.3.11
(Polemikou et al., 2019) Polemikou, A., Zartaloudi, E., & Polemikos, N. (2019). Development of the Greek version of the Spiritual Intelligence Self-Report Inventory-24 (KAPN): factor structure and validation. *Mental Health, Religion and Culture*, 22(10), 1033–1047. https://doi.org/10.1080/13674676.2019.1692195
(Polemikou & Vantarakis, 2019) Polemikou, A., & Vantarakis, S. (2019). Death anxiety and spiritual intelligence as predictors of dissociative posttraumatic stress disorder in Greek first responders: A moderation model. *Spirituality in Clinical Practice*, 6(3), 182–193. https://doi.org/10.1037/scp0000203
(Rahmanian et al., 2019) Rahmanian, M., Hojat, M., Fatemi, N., Mehran, A., & Parvizy, S. (2019). Spiritual intelligence of adolescents with diabetes based on demographic components. *Journal of Education and Health Promotion*, 8(1). https://doi.org/10.4103/jehp.jehp_361_18

(Sharif et al., 2019) Sharif, S., Nia, H., Lehto, R. H., Moradbeigi, M., Naghavi, N., Goudarzian, A. H., Yaghoobzadeh, A., & Nazari, R. (2019). The Relationship Between Spirituality Dimensions and Death Anxiety among Iranian Veterans: Partial Least Squares Structural Equation Modeling Approach. *Journal of Religion and Health*. https://doi.org/10.1007/s10943-019-00931-6
(Sharma, 2019) Sharma, S. (2019). A co-relational study of adjustment among professional and non-professional working women with respect to psychological variables. *International Journal of Recent Technology and Engineering*, 8(1C2), 230–238.
(Abdollahpour & Khosravi, 2018) Abdollahpour, S., & Khosravi, A. (2018). Relationship between spiritual intelligence with happiness and fear of childbirth in iranian pregnant women. *Iranian Journal of Nursing and Midwifery Research*, 23(1), 45–50. https://doi.org/10.4103/ijnmr.IJNMR_39_16
(Antunes et al., 2018) Antunes, R., Silva, A. P., & Oliveira, J. (2018). Spiritual Intelligence Self-Assessment Inventory: Psychometric properties of the Portuguese version of SISRI-24. *Journal of Religion, Spirituality and Aging*, 30(1), 12–24. https://doi.org/10.1080/15528030.2017.1324350
(Baloochi et al., 2018) Baloochi, A., Abazari, F., & Mirzaee, M. (2018). The relationship between spiritual intelligence and aggression in medical science students in the southeast of Iran. *International Journal of Adolescent Medicine and Health*, 32(3). https://doi.org/10.1515/ijamh-2017-0174
(Fazlolah, 2018) Fazlolah, M. (2018). An investigation of the relationship between identity and spiritual intelligence of second-grade high school teachers of the city of Khorramabad. *Annals of Tropical Medicine and Public Health*, 14(Special Is), S743.
(Mahmood et al., 2018) Mahmood, A., Arshad, M. A., Ahmed, A., Akhtar, S., & Khan, S. (2018). Spiritual intelligence research within human resource development: a thematic review. *Management Research Review*, 41(8), 987–1006. https://doi.org/10.1108/MRR-03-2017-0073
(Malini & Raju, 2018) Malini, S., & Raju, R. (2018). Exploring the Moderating Effects of Gender on the Relationship between Motivation and Spiritual Intelligence of Higher Education Students in India. *International Journal of Educational Sciences*, 21(1–3), 79–90. https://doi.org/10.31901/24566322.2018/21.1-3.1056
(Rahmanian et al., 2018) Rahmanian, M., Hojat, M., Jahromi, M., & Nabiolahi, A. (2018). The relationship between spiritual intelligence with self-efficacy in adolescents suffering type 1 diabetes. *Journal of Education and Health Promotion*, 7(1), 100. https://doi.org/10.4103/jehp.jehp_21_18
(Riahi et al., 2018) Riahi, S., Goudarzi, F., Hasanvand, S., Abdollahzadeh, H., Ebrahimzadeh, F., & Dadvari, Z. (2018). Assessing the Effect of Spiritual Intelligence Training on Spiritual Care Competency in Critical Care Nurses. *Journal of Medicine and Life*, 11(4), 346–354. https://doi.org/10.25122/jml-2018-0056

(Zarrinabadi et al., 2018) Zarrinabadi, Z., Isfandyari-Moghaddam, A., Erfani, N., & Tahour Soltani, M. (2018). The codification of spiritual intelligence measurement model in librarianship and medical information science students of medical universities in Iran. *Journal of Education and Health Promotion*, 7(1), 59. https://doi.org/10.4103/jehp.jehp_78_17
(Udin et al., 2017) Udin, Handayani, S., Yuniawan, A., & Rahardja, E. (2017). Antecedents and consequences of affective commitment among Indonesian engineers working in automobile sector: An investigation of affecting variables for improvement in engineers role. *International Journal of Civil Engineering and Technology*, 8(10), 70–79.
(Amirian & Fazilat-Pour, 2016) Amirian, M.-E., & Fazilat-Pour, M. (2016). Simple and Multivariate Relationships Between Spiritual Intelligence with General Health and Happiness. *Journal of Religion and Health*, 55(4), 1275–1288. https://doi.org/10.1007/s10943-015-0004-y
(Chan & Siu, 2016) Chan, A. W. Y., & Siu, A. F. Y. (2016). Application of the Spiritual Intelligence Self-Report Inventory (SISRI-24) among Hong Kong university students. *International Journal of Transpersonal Studies*, 35(1), 1–12. https://doi.org/10.24972/ijts.2016.35.1.1
(Geram, 2016)Geram, K. (2016). The Prediction of Social Adjustment Based on Emotional and Spiritual Intelligence. *International Journal of Advanced Biotechnology and Research*, 7(4), 19–25.
(Jorge et al., 2016) Jorge, D. F. O., Esgalhado, G., & Pereira, H. (2016). Inteligência espiritual: Validação preliminar da versão portuguesa da Escala de Inteligência Espiritual Integrada (EIEI). *Analise Psicologica*, 34(3), 325–337. https://doi.org/10.14417/ap.982
(Anwar & Gani, 2015) Anwar, A., & Gani, A. M. O. (2015). The effects of spiritual intelligence and its dimensions on organizational citizenship behaviour. *Journal of Industrial Engineering and Management*, 8(4), 1162–1178. https://doi.org/10.3926/jiem.1451
(Mahasneh et al., 2015) Mahasneh, A. M., Shammout, N. A., Alkhazaleh, Z. M., Al-Alwan, A. F., & Abu-Eita, J. D. (2015). The relationship between spiritual intelligence and personality traits among jordanian university students. *Psychology Research and Behavior Management*, 8, 89–97. https://doi.org/10.2147/PRBM.S76352
(Khosravi & Nikmanesh, 2014) Khosravi, M., & Nikmanesh, Z. (2014). Relationship of spiritual intelligence with resilience and perceived stress. *Iranian Journal of Psychiatry and Behavioral Sciences*, 8(4), 52–56. http://www.ncbi.nlm.nih.gov/pubmed/25798174%0Ahttp://www.pubmedcentral.nih.gov/articlerender.fcgi?artid=PMC4364477

(Nekouei et al., 2014) Nekouei, Z. K., Yousefy, A., Neshat-Doost, H. T., Manshaee, G., & Sadeghei, M. (2014). Structural model of psychological risk and protective factors affecting on quality of life in patients with coronary heart disease: A psychocardiology model. *Journal of Research in Medical Sciences*, 19(2), 90–98.
(Saeedi et al., 2014) Saeedi, N. R., Yekta, M., & Masoudi, S. (2014). The relation between spiritual intelligence and religion orientation in students. *Advances in Environmental Biology*, 8(9), 816–820.
(Azizi & Zamaniyan, 2013) Azizi, M., & Zamaniyan, M. (2013). The relationship between spiritual intelligence and vocabulary learning strategies in EFL learners. *Theory and Practice in Language Studies*, 3(5), 852–858. https://doi.org/10.4304/tpls.3.5.852-858
(Azizollah et al., 2013) Azizollah, A., Maede-Sadat, R., Narges, M., & Shekoofeh-Sadat, R. (2013). Relationship between different types of intelligence and student achievement. *Life Science Journal*, 10(SUPPL. 7), 128–133.
(Hassan & Shabani, 2013) Hassan, S. A., & Shabani, J. (2013). The Mediating Role of Emotional Intelligence between Spiritual Intelligence and Mental Health Problems among Iranian Adolescents. *Psychological Studies*, 58(1), 73–79. https://doi.org/10.1007/s12646-012-0163-9
(Kaur et al, 2013) Kaur, D., Sambasivan, M., & Kumar, N. (2013). Effect of spiritual intelligence, emotional intelligence, psychological ownership and burnout on caring behaviour of nurses: A cross-sectional study. *Journal of Clinical Nursing*, 22(21–22), 3192–3202. https://doi.org/10.1111/jocn.12386
(Rani et al., 2013) Rani, A. A., Abidin, I., Rashid, A. H., Rashid, M., & Hamid, A. (2013). The Impact of Spiritual Intelligence on Work Performance: Case studies in Government Hospitals of East Coast of Malaysia. *The Macrotheme Review*, 2(3), 46–59. http://macrotheme.com/yahoo_site_admin/assets/docs/7RaniMR23.40131338.pdf
(Shahbakhsh & Moallemi, 2013) Shahbakhsh, B., & Moallemi, S. (2013). Spiritual Intelligence, Resiliency, and Withdrawal Time in Clients of Methadone Maintenance Treatment. *International Journal of High Risk Behaviors and Addiction*, 2(3), 132–135. https://doi.org/10.5812/ijhrba.11308
(Babanazari, 2012) Babanazari, L., Askari, P., & Honarmand, M. M. (2012). Laleh Babanazari, Parviz Askari, Mahnaz Mehrabizade Honarmand. Spiritual Intelligence and Happiness for Adolescents in High School. *In Life Science Journal* (Vol. 9, Issue 3).
(King et al., 2012) King, D. B., Mara, C. A., & deCicco, T. L. (2012). Connecting the spiritual and emotional intelligences: Confirming an intelligence criterion and assessing the role of empathy. *International Journal of Transpersonal Studies*, 31(1), 11–20. https://doi.org/10.24972/ijts.2012.31.1.11

(Narayanan & Jose, 2011) Narayanan, A., & Jose, T. P. (2011). Spiritual intelligence and resilience among christian youth in Kerala. *Journal of the Indian Academy of Applied Psychology*, 37(2), 263–268.
(King & deCicco, 2009) King, D. B., & de Cicco, T. L. (2009). A Viable Model and Self-Report Measure of Spiritual Intelligence. *The International Journal of Transpersonal Studies*, 28(1), https://doi.org/68–85. 10.24972/ijts.2009.28.1.68
(Martin & Hafer, 2009) Martin, T. N., & Hafer, J. C. (2009). Models of emotional intelligence, spiritual intelligence, and performance: A test of tischler, biberman, and mckeage. *Journal of Management, Spirituality and Religion*, 6(3), 247–257. https://doi.org/10.1080/14766080903069364
(Yang & Wu, 2009) Yang, K.-P., & Wu, X.-J. (2009). Spiritual intelligence of nurses in two Chinese social systems: A cross-sectional comparison study. *Journal of Nursing Research*, 17(3), 189–198. https://doi.org/10.1097/JNR.0b013e3181b2556c
(Yang & Mao, 2007) Yang, K.-P., & Mao, X.-Y. (2007). A study of nurses' spiritual intelligence: A cross-sectional questionnaire survey. *International Journal of Nursing Studies*, 44(6), 999–1010. https://doi.org/10.1016/j.ijnurstu.2006.03.004
(Tirri et al., 2005) Tirri, K., Ryhänen, T., & Nokelainen, P. (2005). The Intelligence Profile of Finnish Peacekeepers. *Gifted and Talented International*, 20(2), 19–30. https://doi.org/10.1080/15332276.2005.11673450

Table S2. Spiritual Intelligence areas of application analysis.

Areas of Application	Frequency
Adaptative Cognition	164
Critical contemplation of purpose, meaning and values	53
Drawing meaning and purpose from all physical and mental experiences	24
Adjustment	17
Creation and mastering of life purpose	15
Refrain from conditioning from social norms and stereotypes	8
Creativity	8
Reframe experiences	6
Abstract thinking	5
Elevated Consciousness	98
Sanctification of daily-life activities	29
Capacity to transcend the physical and material world	27
Realization of multidimensional reality	26
Ability to experience heightened states of consciousness	15
Problem Management	92
Holistic problem-solving	35
Overcoming difficulties using spiritual resources	28
Finding solutions to life problems	16
Achieving goals	13
Personal Growth	61
Connecting inner-self to life-world experiences	16
Pursuit of personal well-being	16
Positive self-concept	14
Achieving internal and external integrity and consistency	10
Humanitarian Principles	51
Ethical behavior	16
Acts of compassion	8
Wise behavior	7
Attitude of humility	4
Existentialism	35
Critically contemplate existential or metaphysical issues	32
Existentialism	3
Mental Health	30
Reduce stress and burnout	11
Reduce mental illness	11
Reduce depression	3
Emotional healing	3
Interpersonal Relationships	28
Being empathetic	10
Being accountable in interpersonal relationships	6
Using intelligence to serve others	5
Develop trust in others	4
Work Performance	20
Work integrity and independence	7
Self-motivation	5
Holistic care	5
Sense of achievement	2

Footnote: Frequency refers to the overall number of times the occurrence was mentioned in all the 112 included articles.

Table S3. Spiritual intelligence surrogate terms analysis.

Surrogate Terms		Frequency
Existential Intelligence		13
	Existential Intelligence	4
	Moral Intelligence	3
	Soul's Intelligence	2
	Fundamental Intelligence	1
	Ultimate Intelligence	1
	Spiritual Intellect	1
	Wisdom Intelligence	1
Spiritual Well-being		7
	Spiritual Well-being	5
	Spiritual Strength	1
	Spiritual Coping	1
Spiritual Quotient		5
Heightened Consciousness		4
	Heightened Consciousness	1
	Abstract Reasoning	1
	Spiritual Consciousness	1
	Spiritual Experiences	1

Footnote: Frequency refers to the overall number of times the occurrence was mentioned in all the 112 included articles.

Table S4. Spiritual intelligence related terms analysis.

Related Terms	Frequency
Spiritual Experience	142
Spirituality	50
Religious Beliefs	35
Spiritual Well-being	15
Spiritual Growth	11
Spiritual Leadership	4
Health	115
Mental Health	30
Well-being	23
Stress Coping and Emotional Regulation	16
Resilience	12
General Health	8
Quality of Life	7
Performance	72
Patient Care	44
Transformative Education	19
Work Performance	10
Leadership	8
Human Resources	5
Humanitarian Principles	57
Ethics and Moral Values	18
Empathy and Compassion	9
Happiness	6
Motivation	5
Cognition	59
Emotional Intelligence	26
Existential Intelligence	9
Rational Intelligence	7
Multiple Intelligences	4
Self-consciousness	49
Meaning and Purpose	10
Holism	8
Self-management and Efficacy	6
Mindfulness	5
Organizational Development	16
Organizational Behavior	5
Work Environment	5
Organizational Spirituality	2
Interpersonal Relationships	10
Human Relationships and Communication	4
Social Adjustment	3
Social System	2

Footnote: Frequency refers to the overall number of times the occurrence was mentioned in all the 112 included articles.

Table S5. Spiritual intelligence antecedents' analysis.

Antecedents	Frequency
Spiritual-self	55
Spiritual Self-awareness	25
Spiritual Experiences	12
Transcendental Integration	6
Spirituality	5
Awareness	29
Holistic View	12
Self-consciousness	9
Transcendental Awareness	5
Existentialism	15
Existential Questioning	9
Reflective Activities	4
Meaning and Value	11
Creating Meaning and Purpose	7
Integrative Organizing Principles and Values	4
Self-investment	9
Commitment to Self-care and Knowledge	7
Search for Wholeness	2
Emotional-self	8
Emotional Growth and Awareness	5
Empathy	3
Cognition	7
Inner Resources Perception	6
Humanitarian Traits	5
Forgiveness and Humility	2

Footnote: Frequency refers to the overall number of times the occurrence was mentioned in all the 112 included articles.

Table S6. Spiritual intelligence consequents' analysis.

Consequents	Frequency
Better Health	145
Reduced stress, anxiety and burnout	40
Better psychological adjustment and emotional regulation	34
Better mental health	27
Higher Quality of Life	12
General health	12
Better self-care and self-concept	10
Satisfaction in life	9
Better Work Performance	93
Better caring behavior	23
Better job performance	19
Increased self-efficacy and productivity	16
Higher job satisfaction	12
Academic eagerness	4
Humanitarian Orientation	74
Appropriate behavior	21
Resilience	19
Happiness	14
Flexibility	9
Motivation	4
Higher Self-conscience	40
Self-awareness	11
Holistic attitude and sense of wholeness	9
Ability to overcome hardships of life	8
Self-fulfilment and growth	5
Meaning and Value in Life	22
Meaningful life and relationships	13
Purposeful life	4
Meaningful work	3
Better Organizational Development	22
Organizational commitment	8
Organizational citizenship behavior	6
Organizational performance	5
Organizational development	3
Better Interpersonal Relationships	27
Better social adjustment	9
Better communication	9
Mutual understanding and empathetic relationships	4
Higher Cognition	12
Higher emotional intelligence	7
Holistic problem-solving	5
Higher intellectual development	4
Higher human performance	3
Higher Spiritual Experience	8
Transcendent spiritual states	3
Desire for religious experiences	2

Footnote: Frequency refers to the overall number of times the occurrence was mentioned in all the 112 included articles.

Table S7. Spiritual intelligence attributes' analysis.

Attributes	Frequency
Equanimity	232
Integrity	62
Calm and balanced	39
Adaptability and resilience	38
Body-mind wholeness	32
Moral and ethical behavior	26
Optimism and happiness	20
Creativity	15
Life Wisdom	196
Humanitarian conduct	105
Interpersonal harmony	57
Personal achievement	34
Transcendental Awareness	219
Transcendental awareness	85
Multiple levels of consciousness	76
Holistic thinking	47
Daily-life sanctification	11
Meaning and Purpose Creation	114
Meaning creation	77
Sense of purpose	12
Gratitude	9
Life goals	9
Motivation	7
Spiritual Consciousness	108
Higher cognition	51
Spiritual maturity	28
Immanence	17
Spiritual experiences	12
Existential Questioning	91
Deep understanding of existential questions	62
Self-knowlegde	15
Intellectual pursuit of knowledge	14

Footnote: Frequency refers to the overall number of times the occurrence was mentioned in all the 112 included articles.
